# Making parks make a difference: poor alignment of policy, planning and management with protected-area impact, and ways forward

**DOI:** 10.1098/rstb.2014.0280

**Published:** 2015-11-05

**Authors:** Robert L. Pressey, Piero Visconti, Paul J. Ferraro

**Affiliations:** 1 Australian Research Council Centre of Excellence for Coral Reef Studies, James Cook University, Townsville, Queensland 4811, Australia; 2 Microsoft Research, Computational Science Laboratory, 21 Station Road, Cambridge CB1 2FB, UK; 3 Carey School of Business and Department of Geography and Environmental Engineering, Whiting School of Engineering, Johns Hopkins University, Baltimore, MD 21218, USA

**Keywords:** policy targets, conservation planning, performance management, impact evaluation, saving biodiversity

## Abstract

Policy and practice around protected areas are poorly aligned with the basic purpose of protection, which is to make a difference. The difference made by protected areas is their impact, defined in program evaluation as the outcomes arising from protection relative to the counterfactual of no protection or a different form of protection. Although impact evaluation of programs is well established in fields such as medicine, education and development aid, it is rare in nature conservation. We show that the present weak alignment with impact of policy targets and operational objectives for protected areas involves a great risk: targets and objectives can be achieved while making little difference to the conservation of biodiversity. We also review potential ways of increasing the difference made by protected areas, finding a poor evidence base for the use of planning and management ‘levers’ to better achieve impact. We propose a dual strategy for making protected areas more effective in their basic role of saving nature, outlining ways of developing targets and objectives focused on impact while also improving the evidence for effective planning and management.

## 1. Introduction

The primary role of protected areas is nature conservation [1]. Nature conservation is easily interpreted as intervening in the loss of ecosystems,^1^ species and other valued aspects of the natural environment. It follows that the success of protected areas should be measured in terms of how much loss has been avoided. This is, after all, what people mean when they talk about saving biodiversity. Avoided loss is referred to as impact. Adapting a definition from the well-established field of programme evaluation [2], impact is the difference that protected areas make to one or more intended (or unintended) outcomes, relative to the counterfactual [3] of no intervention or a different intervention [4,5]. So impact is the difference between what we see in a protected area and what we would see there if it had not been established. If the counterfactual is much worse for nature conservation, then protection has had a large impact.

The basic purpose of protected areas in avoiding loss might seem straightforward, but it presents difficulties for policy-makers and practitioners. One difficulty is that little is known about how much difference most protected areas actually make [4]. This is in strong contrast to fields such as medicine, education and development aid, in which programme evaluation is firmly established [6,7]. A second difficulty is that many protected areas have not been located, configured or managed to maximize their impact. Protected areas tend to be residual [8] in the sense of covering parts of the land [9–11] and sea [12] with least potential for extractive activities. This residual tendency means that they are, in effect, located to have little impact [13–16]. A third difficulty is that quantitative policy targets [17] and operational objectives for planning and managing protected areas [18,19] are not framed in terms of impact, and could be achieved without contributing to impact. Taken together, these problems indicate a large gap between the primary role of protected areas and current policy and practice.

The remainder of this paper elaborates on these problems and proposes ways forward. Section 2 is an extended problem statement. It covers commonly used and highly influential policy targets and objectives for planning and management, and measures of progress towards them. It reviews the information provided by five types of measures and explains how they fail to provide information on impact and so risk misdirecting limited conservation resources. Section 3 explores how some of the ‘levers' that could be manipulated by protected-area planners and managers might be better directed towards impact. One finding of this section is the weak evidence to link some of these levers to impact and to guide practitioners in using levers in combination. Section 4 proposes some ways forward, suggesting how policy targets and operational objectives might be recast to focus them on impact, while concurrently improving the evidence base to guide practitioners. This new focus on impact can apply globally, but also to planning contexts varying in scope from national or regional to local. Section 5 summarizes the challenges and opportunities involved in refocusing policy and practice on avoiding loss. Throughout the article, we discuss impact in relation to biodiversity, although it can also be framed in terms of other intrinsic natural values, ecosystem services, livelihoods, and other socioeconomic considerations.

## 2. Impact relative to other measures of protected-area performance

Many terms in conservation policy, science and practice are meant to describe the goals and performance of protected areas. Some terms are used interchangeably [20,21], even when they relate to different concepts and require different approaches to measurement.

This article draws on four areas of activity related to protected areas – formulation of policy, conservation planning, performance management, and impact evaluation – each with its own terms and definitions. Table 1 is a glossary to explain the terms we use in the following sections. Sections 2*a*,*b* clarify the relationships between terms used for protected-area policy targets, operational objectives, and measures of progress, and explain the potential for commonly used measures to obscure rather than reveal the impact of protected areas.

**Table 1.** Glossary of important terms.

policy	
targets	qualitative or quantitative aspirational statements about protected-area achievements, such as the Convention on Biological Diversity's Aichi targets [17]
measures	quantitative ways of stating policy targets or gauging progress towards them (examples are in figure 1)

conservation planning	
goals	high-level, qualitative statements about the intended consequence(s) of conservation actions [22]
objectives	more specific statements than goals, expressed quantitatively, that interpret goals through the filter of available information [22], relating here to operational decisions about planning and management of protected areas
actions	protected areas themselves or types of protective management or restoration, both within and outside protected areas [23,24], equivalent to attributes in impact evaluation (below)
measures	quantitative ways of stating objectives or gauging progress towards them (examples are in figure 1)

performance management	
inputs	investments in protected-area programs or the raw materials for actions related to protected areas [25]
outputs	the concrete, countable products of one or more conservation actions [25]
outcomes	the assumed short- and medium-term effects of an intervention's outputs [25], further defined here as measured only within protected areas (figure 2)
assumptions	hypotheses about factors that could affect the progress or success of actions, made explicit in theory-based evaluations that systematically track anticipated results chains [25]

impact evaluation	
impact	the value added to a counterfactual estimate of a variable of conservation interest [3,4]
attributes	actions (see above), described by type and amount, that define protected-area treatments [26]
treatments	forms of protection and approaches to management, defined by attributes [26]
mechanisms	in this article, threats to biodiversity, affected by treatments [26]
moderators	variables not affected by treatments but modifying the impact arising from treatments [26]
assumptions	hypotheses about factors that could affect the progress or success of attributes (actions) and treatments, made explicit in theory-based evaluations that systematically track paths to impact [7]

### (a) Terms and definitions

**Figure 1.**
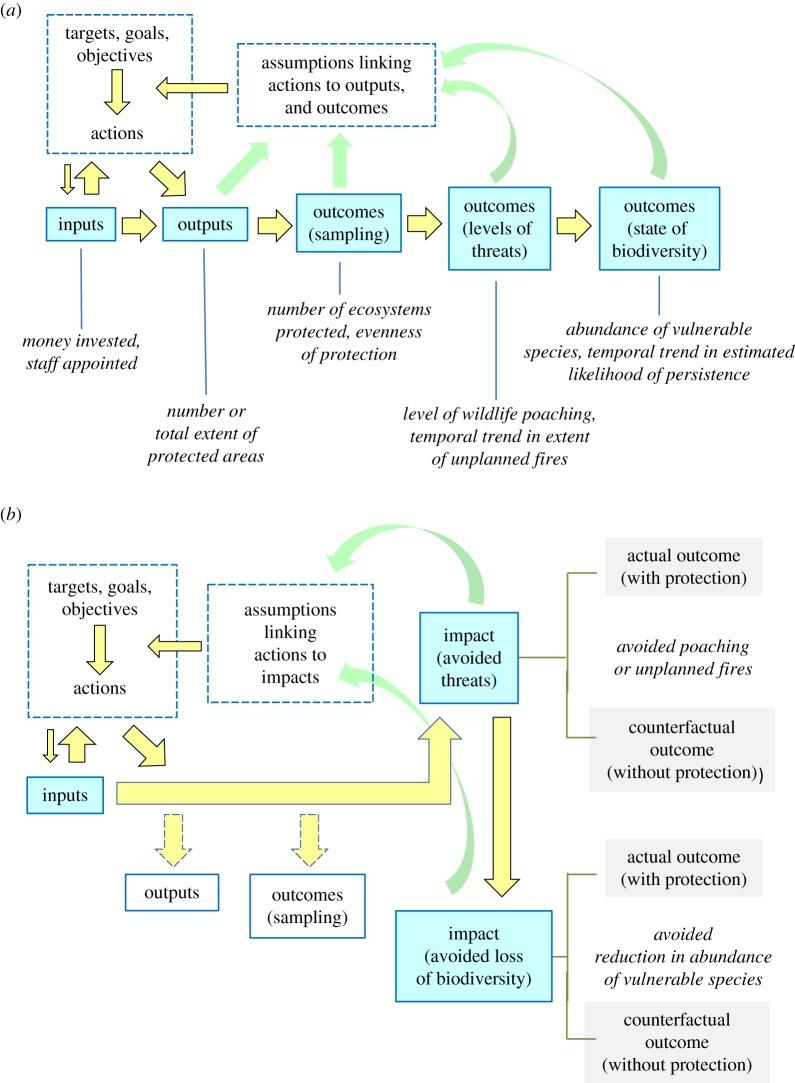
Ways of achieving and measuring progress towards biodiversity conservation through protected areas. Blue boxes are types of measures used in performance management (*a*) or types of impact estimated from counterfactual analyses (*b*). Yellow arrows indicate influence. Terms in italics are examples of ways of setting specific targets and objectives or measuring progress towards them. (*a*) Results chain of inputs, outputs and outcomes, illustrating the business-as-usual approach to protected areas, focused on performance measures that can be misleading about protected-area impact. Types of measures in the results chain concern the extent, content or state of protected areas or temporal trends within them. The green feedback arrows from performance measures to assumptions refer to the recommendation for results chains to be applied adaptively, as achievements are measured [20]. (*b*) Policy targets and protected-area planning and management directed to making a difference. With this model, outputs and outcomes for sampling are incidental, achieved as means to the end of impact in terms of avoided threats or (preferably) avoided loss of biodiversity. The green arrows returning to assumptions indicate that impact evaluation feeds evidence back into programme design for learning and adaptive decision-making [4,21]. Definitions of terms in dashed boxes are in table 1.

Figure 1 is deliberately simple and generic, the better to illustrate types of measures and highlight that many are not informative about protected-area impact. The sequence of boxes in figure 1*a* describes a results chain [20,27] or logic model [21], which is one way of laying out a basic theory of change^2^ [7] to guide conservation interventions. For the five steps in the results chain—from inputs to outcomes related to the state of biodiversity—we adapted the terms (see table 1), definitions and sequence from several sources [20,21,28–30].

**Figure 2.**
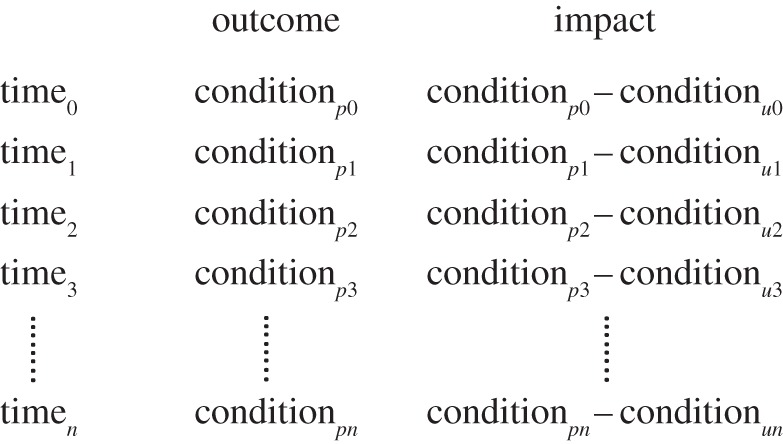
Distinguishing outcomes from impacts as defined in this paper. Outcomes are the conditions in protected areas: the content, threat levels, or state of biodiversity within protected areas (*p*) at a point in time (e.g. condition*_p_*_2_) or at multiple points in time, which reveal temporal trends. Impacts are the differences between conditions at sites within protected areas (*p*) and estimates of the conditions at the same sites were protection not present (*u*), or the counterfactual conditions. Ideally, impacts are also estimated at multiple points in time to test for differences in trends within and outside protected areas. The reliability of impact estimates varies with study design and quantitative rigour [14].

A potential difficulty for some readers is the definition of ‘impact’ that applies here (table 1). In §1, we defined the term as the value added to a counterfactual estimate of some variable of conservation interest [3]: the difference that protection makes relative to the estimate expected without protection (figure 2). Alternative definitions have some currency, so figure 1 compares two lines of thinking [31]: process evaluations for performance management (figure 1*a*) and frameworks for counterfactual analyses (figure 1*b*). In performance management (figure 1*a*), ‘impact’ has a different meaning from the one used here: ‘Positive and negative, primary and secondary long-term effects produced by a[n] … intervention, directly or indirectly, intended or unintended’ [25, p. 24], ‘desired end goals of the project’ [20, p. 3], 'the desired future state of a conservation target' [27, p. 39] and ‘lasting improvement[s] in the conservation status of biodiversity … ’ [32, p. 2]. We refer to these performance measures as outcomes when they are estimated only within protected areas (figure 2). A key message in figure 1, then, is that impact is not part of a results chain defined for performance management.

We discuss impact only in relation to avoided threats and (particularly) avoided loss of biodiversity (figure 1*b*). These are the most important for protected-area policy targets and operational objectives for planning and management. In principle, though, inputs, outputs and outcomes (figure 1*a*), if they are mechanisms through which protected areas operate, could have associated counterfactual estimates. For example, non-spatial interventions, such as high-level political lobbying, could enlarge the supply of funds to establish protected areas in a region, thereby having a positive impact on inputs and potentially leading to larger impacts for biodiversity.

### (b) Five types of measures in the results chain

Our aim with figure 1 is to produce a broad taxonomy that allows any target, objective or measure to be understood relative to impact. We expand on the five types of performance measures in the results chain (figure 1*a*) to demonstrate how they can fail to provide information about impact, with the risk of misdirecting resources for conservation. We acknowledge the value of results chains in laying out assumed causal linkages between interventions and desired results [20], especially for outcomes reliant on management of established protected areas. In §3*b*(i), we call for similar theories of change to guide practitioners in achieving impact; but focusing on impact requires counterfactual thinking (figure 1*b*), which is typically absent from results chains for performance management [21].

#### (i) Inputs

Inputs are investments in protected-area programmes or the raw materials for a programme or project [30], usually consisting of time and money [28] for staff and actions [25]. Ideally, targets, or goals and objectives, then actions (table 1) would be identified first to determine required inputs (figure 1), but inputs are more commonly determined pre-emptively according to available budgets and competing demands on resources. Consequently, inputs tend to constrain potential actions (figure 1). The value of inputs depends on their ultimate influence on impacts. This influence might be diluted or neutralized if attention remains focused only on steps in the results chain of figure 1*a*. Other reasons for the lack of connection between inputs and impact [4,33,34] include: the difficulty of collecting accurate data on inputs, with accounting and budgets often fragmented across operational units in organizations; lack of goals for impacts; lack of data on impacts; political motivations for spending as an end in itself, unconnected to outcomes or impacts, to demonstrate commitment to vaguely defined conservation goals; and constraints on where and how funds can be spent effectively.

#### (ii) Outputs

Outputs are the concrete, countable products of one or more conservation actions [25]. Outputs have also been described as implementation [28]. Common examples of outputs are numbers or total extent of protected areas, or percentages of jurisdictions or regions protected. Measures of outputs are attractive because they can be estimated with relative ease and certainty, but outputs fail to inform planners, funders and policy-makers about the limitations of protected areas, including ineffective management and poor compliance.

Outputs are uninformative about the contributions of protected areas to conservation outcomes [35,36], and outcomes can be overlooked when targets and related reporting are focused on outputs [37]. In particular, outputs obscure the general tendency for protected areas to be residual to extractive uses [9–11], so outputs can greatly overstate impact. Across regions, differences in protected-area extent might simply reflect differences in the amount of unproductive land [10]. Over time, expansion of protected-area systems can be associated with decreasing impact, reflected in increasing bias of protection away from ecosystems and species most in need of conservation interventions [38]. Policy targets framed as outputs are essentially arbitrary, and disconnected from the requirements for persistence of biodiversity [39,40]. Importantly, undue attention to outputs, often encouraged by poorly considered policy targets, could be counterproductive for achieving impacts. Seeking outputs alone could, for example, motivate establishment of extensive protected areas in remote areas where avoided losses of biodiversity are small, or divert resources from more appropriate and effective conservation approaches [41,42]. Pursuing the policy target of 17% protected-area coverage globally, in the absence of species-specific objectives for avoided loss, risks having negligible or even negative impact on threatened species [43].

#### (iii) Outcomes for sampling

Generally, outcomes are the assumed short- and medium-term effects of an intervention's outputs [25]. For conservation, outcomes are the effects of a project on the conservation problem of interest [28] or interim results achieved by outputs [20]. Sampling outcomes reflect the extent to which aspects of biodiversity—potentially defined by features such as ecosystems, species or genotypes—are represented within protected areas. Sampling is directly equivalent to measures of representativeness [44], and to a key principle of systematic conservation planning—representation [45]. Ways of measuring sampling outcomes include coverage of features by generic [46] or multiple-action [24] protected areas, evenness of representation [47] and modelled trade-offs between biodiversity conservation and competing goals [48].

Sampling is the most immediate and easily measured outcome in the results chain and the main focus of systematic conservation planning [18], but sampling is also the outcome that is least informative about impact because of several limitations (aside from its lack of relationship with persistence [45,49]). First, reporting on or targeting sampling of extensive features such as biomes and ecoregions [50,51] fails to account for their physical and biological heterogeneity, usually associated with heterogeneity of costs and threats. Consequently, increasing representation or reaching targeted levels of these features can be achieved through formal protection of residual areas within them [10]. Such protection exacerbates biases away from biodiversity most in need of protection and contributes little to impact. For the same reason, minimizing the costs of representing extensive, heterogeneous features comes with the potentially perverse result of directing protection away from areas with distinctive species and ecosystems that are highest in cost but also most in need of intervention [12].

A second limitation of sampling outcomes is that they indicate nothing about the relative urgency of protection. Even informed by objectives scaled to threat and other aspects of relative need [40], an increase in representativeness over a defined period does not indicate whether protection has been afforded to features requiring it most urgently because they are in rapid decline, or simply to features that were easiest to protect (and probably, therefore, not urgently needing protection). The likelihood of the second possibility is indicated by the general residual pattern of protected areas [9]. Indeed, longitudinal studies have shown that representativeness can increase while bias away from ecosystems most in need of protection also increases [38] or remains unchanged [52]. Urgency is a key consideration in the many situations in which conservation actions need to be scheduled, not just located spatially, to achieve impact. Examples of simultaneous application of protection across entire portfolios of areas [53,54], although feted as success stories, tend to be outliers. The greatest conservation challenges are in situations, such as inshore marine waters in developing countries and private land in most countries, where limits on resources and the need to negotiate protection demand incremental actions over extended periods while biodiversity loss continues [55]. Representation alone is inadequate to guide effective scheduling strategies for incremental protection [18].

**Figure 3.**
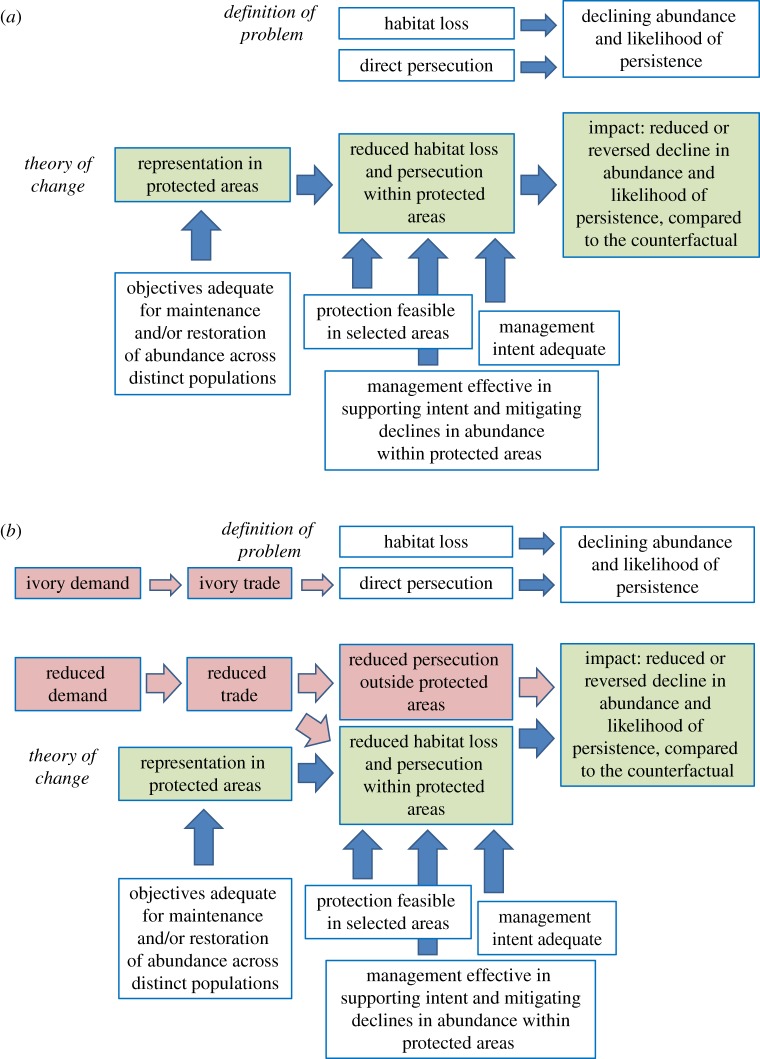
Theories of change for achieving impact for two mammal species. (*a*) Red wolf, *Canis rufus.* (*b*) African elephant, *Loxodonta africana.* Red boxes in (*b*) indicate additional important considerations for the African elephant. These theories of change are simplified to support key points in the text and to illustrate differences between species. More elaborate and informative theories of change are preferable for guiding conservation interventions [20], ideally using the causal inference framework of Ferraro and Hanauer [26] shown in figure 4.

A third limitation of sampling outcomes is that the theory of change [7,20,26] related to representation is simplistic. The theory assumes that coverage by protected areas is equivalent to removal of threats. If this assumption does not hold, then priorities for representation [46,56] could be unrelated to those for impact. Even for imperilled species, and even for those heavily reliant on protected areas, representation does not necessarily lead to impact. The red wolf, *Canis rufus*, for example, has a limited range and faces threats that can be mitigated by protected areas; but, even in this case, the typical theory of change for representation (green boxes in figure 3*a*) is naive, implying that representation per se is sufficient. For this species, representation could lead to impact, subject to conditions, only some of which are shown in figure 3*a*: objectives for sampling being adequate to promote the persistence of the species, including genetically and geographically distinct populations that might be facing different levels of threats; feasibility of protection in the selected areas; appropriate management intent (interpreted in §3 as allowed and regulated uses); and adequate management effectiveness. With these conditions met, impact would be reflected in abundances of red wolf on more stable or steeply rising trajectories within protected areas compared with the estimated trajectories in the absence of protection.

For other imperilled species, both the typically simple and slightly elaborated theories of change in figure 3*a* are much less relevant. The African elephant, *Loxodonta africana*, for example, has an extensive range, low density and rapid rate of decline. The key threat to this species is demand for ivory, on which protected areas have limited direct effect (figure 3*b*). The survival of this species will depend also on reduced demand, trade and hunting, both within and outside protected areas. The unavoidable need for off-park interventions related to markets, transport of ivory and hunting will not be indicated by priorities for representation, and might differ spatially from those priorities. This third limitation of sampling outcomes will be exacerbated in extensive, coarse-resolution assessments for representation that use unrefined distribution data such as range maps [57] and ignore spatial variation in suitability or abundance, varying life-history requirements and likelihood of persistence.

#### (iv) Outcomes for levels of threats

As outcomes, threat levels reflect the influence of protection on the conservation problem of interest [28]. They can be viewed as interim results achieved by outputs [20], with protection and associated management actions intended to reduce the extent or intensity of threats to conservation features of concern. Approaches to measuring outcomes for threats include threat reduction assessment [58] and biases in protection relative to threat [38,52].

Assuming that relevant threats have been identified, focusing attention on threats has some appeal. Protection's effect on biodiversity can take many years to manifest, often beyond the lifetimes of projects [26,28]. Measures of threat are attractive also when adequate biological data are expensive and provide complicated or ambiguous signals [58]. Threats can be easier and cheaper to observe than biodiversity responses, especially when remote sensing is applicable [59]. Furthermore, measuring threat levels adds information to sampling outcomes by more directly addressing the reasons for protection. Sampling concerns only content, not the levels of threat to which sampled features are exposed.

Threat levels are, however, proxies for conservation success in terms of the state or trends of biodiversity. Their effectiveness as proxies can be uncertain for several reasons (and see [20,58]), including: conservation actions focusing on the wrong threats; levels of threat being poorly aligned spatially with biodiversity features of most concern; removal of one threat allowing others to increase (especially relevant to invasive plants and animals); actions in a protected area being insufficient to stop threats intruding from outside; and unexpectedly large reductions in threats being needed to achieve a required biodiversity state. These uncertainties highlight that measuring threat levels involves, implicitly or explicitly, a theory of change (figure 3), including hypothesized causal links between actions and the desired state of biodiversity [58]. Failures to produce intended biodiversity states by reducing threats are not arguments against addressing threats; instead, these failures call for adaptive thinking in constructing theories of change. Nonetheless, under high levels of uncertainty about threats and their effects, the best strategy to maximize biodiversity benefits might be to maximize sampling outcomes and ignore threats altogether [60].

Putting aside their utility as proxies, outcomes for threat levels have a limitation that is more fundamental to this article: by our definition (figure 2), they are measured only within protected areas and lack explicit counterfactuals. It is assumed that previous levels of threat or trends in those levels will persist in the absence of protection and that any change in threat can be attributed to protection and associated management. Reductions in threats might, however, reflect processes unaffected by protection, such as changes in demand for products; in this case, protected areas might have caused little change for the better. Similarly, lack of change in threats within protected areas could be seen as a failure, but this leaves open the possibility that threats outside protected areas could have increased markedly in the absence of interventions; in this case, the apparently ineffective actions might in fact be highly successful. Measures of outcomes in relation to threat levels can therefore over- or understate the real impacts of protection on threats.

Without explicit attention to counterfactuals, measures of threats as outcomes could misdirect conservation resources. Maximizing outcomes for threats could focus new protected areas on places where threats are most easily mitigated, even if the value added by protection (impact) is small or zero, while neglecting places where serious threats can only be slowed, not stopped, but where the estimated difference relative to places without protection (impact) is large.

#### (v) Outcomes for state of biodiversity

Outcomes related to state of biodiversity are the ‘effects' in the conceptual models of the Cambridge Conservation Forum—‘project-scale changes in the conservation status of target ecosystems, habitats, species or populations' [28, p. 158]—and ‘impacts' in the results chains of performance evaluation—the desired future states of features of conservation concern [27].

Whereas sampling outcomes are typically measured in relation to how well features mapped across whole planning domains are represented in protected areas, measures of outcomes for state of biodiversity, as we define them here, are typically based on detailed observations of individual protected areas, sometimes aggregated to whole protected-area systems. The focus here is on state or trends in terms of extent, condition, abundance or likely persistence of protected features, assessed remotely [59] or from field surveys [35,61].

In this step of the results chain, biodiversity state is observed directly, not assumed or predicted from threat levels [58]. Remotely derived data on biodiversity state can be invaluable [59], as long as their limitations are understood. Remote-sensing data on cover of native vegetation, for example, could be seen as proxies for data such as abundances of species or the composition of species within ecosystems, which rely on field surveys and are therefore more difficult and expensive to collect; but both the amount and configuration of remaining native vegetation can be unreliable predictors of biodiversity variables of ultimate interest [62,63].

As outcomes lacking explicit counterfactuals (figure 2), measures of the state of biodiversity could be misleading about impact. Improving or maintaining species status within protected areas could be taken to indicate success of protection, even if the same trends are occurring in unprotected areas and impact is, in fact, zero. Similarly, a protected area suffering declines in numbers of some species could be viewed as a failure, even if trends in numbers under protection are dramatically better than more serious negative trends without protection.

Conservation resources can also be misdirected if counterfactual biodiversity outcomes are not correctly defined. This risk is illustrated by a measure of conservation progress proposed in Australia [64]. The measure compares the amount of additional protection, say of an ecosystem, with the amount of loss of that ecosystem over a defined period, but the measure can be unrelated to impact. The measure would be strongly positive, for example, if extensive new protection focused on already-secure examples of ecosystems, say on public land immune from vegetation loss, and strongly outweighed loss on other tenures; but this would obscure a net loss, with the protection part of the ratio not reflecting impact but simply a change in management authority. Conversely, the ratio could be negative, indicating a poor result for conservation, if a small amount of protection on tenures allowing agricultural expansion avoided loss but was outweighed by unavoidable loss on those same tenures, but the impact of that small conservation intervention could be larger than in the previous case.

### (c) Are policy, planning and management directed to means or ends?

One limitation of conceptualizing policy targets and operational objectives with the results chain (figure 1*a*) is that maximizing achievement at one step will not necessarily maximize achievement at subsequent ones and might, in fact, compromise them. Maximizing outputs as protected-area extent, for example, will not necessarily maximize outcomes for threat levels or state of biodiversity. This point might seem obvious, but is not reflected in the current focus of quantitative policy targets on outputs [17], the preoccupation of systematic conservation planning with sampling outcomes [18] and the scant attention given to impacts in measures of protected-area management effectiveness [19,65].

The results chain is limited, however, in a more fundamental way: it provides no roadmap to maximizing impact. In §2*b*, we showed that the measures in the results chain of figure 1*a*, from inputs to outputs and three types of outcomes, can be misleading about protected-area impact. Any achievements in terms of impact will necessarily be incidental if impact is not addressed directly, and none of the measures in the results chain takes this direct approach. Impact is the primary role of protected areas, but the incidental, and typically small (maximum 7% of protected-area extent [13–16]) impact achieved so far indicates that a business-as-usual approach to establishing and managing protected areas (figure 1*a*) needs a major and urgent overhaul.

Impact is the end point that all conservation scientists and practitioners seek. The measures in figure 1*a* are possible means to this end, but they are commonly seen as ends in themselves. At worst, they can be achieved with minimal impact, or used perversely to obscure lack of progress towards impact. At best, they are part of a larger suite of measures that also cover impact, in which case the results chain and impact can be reconciled.

Measuring conditions of interest both within and outside protected areas (figure 2) provides information needed for adaptive management and boundary adjustments both to achieve successful outcomes and maximize impact (figure 1*b*). Another article in this theme issue [66] demonstrates the feasibility of such an extended monitoring design, established initially for outcomes, then enlarged for impact. Information for outcomes and impact can be complementary. If, for example, protected areas avoided some loss of a species of concern (impact) but that species was still in decline within the protected-area system (outcome), the need for better management within the system, or different investments outside protected areas, would be indicated (e.g. figure 3*b*). If management outcomes were favourable, but conditions outside protected areas indicated little impact, practitioners might draw lessons about still better management, or about better placement of new protected areas. Achieving impact with new protected areas requires a predictive approach, outlined in §4.

A focus only on measures in the results chain (figure 1*a*) in setting policy targets, planning new protected areas, and managing established ones runs the serious risk of failing to achieve impact. Maximizing the impact of protected areas needs impact-specific policy targets and impact-specific operational objectives for planning and management. These targets and objectives should be based on an understanding of the ways in which protected-area impact can be achieved.

## 3. Ways of achieving protected-area impact

**Figure 4.**
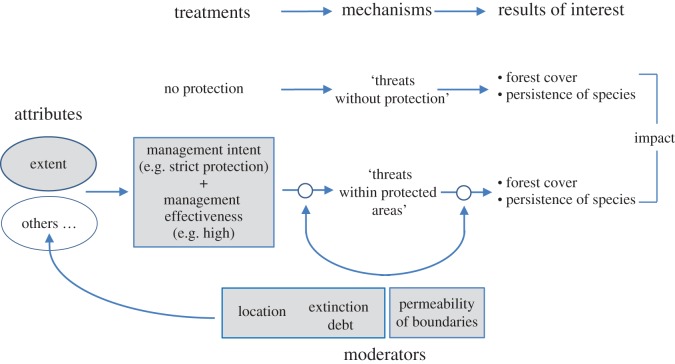
Adaptation of a conceptual diagram (from [26]) of how protected-area impact is influenced by attributes, treatments, mechanisms and moderators. Shading indicates the attribute, treatments and moderators discussed as possible ways for practitioners to achieve impact. The figure is simplified deliberately to focus on these variables.

Here, we describe some of the ways in which policy-makers, planners and managers can influence protected-area impact, and highlight three implications of this analysis for policy and practice. To understand the ‘levers' that might be manipulated (or actions taken) to achieve impact, we adapt a framework presented elsewhere in this theme issue [26] to produce a simplified theory of change (figure 4), giving the examples of forest cover and persistence of species as measures of impact. Our terms here (table 1) are from the literature on program evaluation and causal inference (see [26]).

The utility of the framework in figure 4 is that it clarifies the causal paths between practitioners’ levers or actions on the left and impact on the right. The key points of figure 4 are:

— Attributes are actions (table 2), described by type and amount. Examples are allowed and regulated uses, levels of staffing and funding for management, levels and types of enforcement, ways of controlling invasive plants, and approaches to liaison with neighbouring communities. In some of our examples below, attributes apply outside protected-area boundaries when they are intended to reduce adverse external influences.
— Treatments are the forms of protection and approaches to management, defined by attributes.
— Mechanisms in our example are types and levels of threats to biodiversity, such as density of invasive plants or lack of compliance. Mechanisms lie on a causal path between treatments and impact, representing both intermediate outcomes of treatments and intermediate treatments that directly affect impact.
— Moderators are variables that are unaffected by treatments but modify the amount of impact arising from treatments. Moderators can operate in three ways: by shaping the pre-treatment conditions of places chosen for protection; by altering the effects of treatments on mechanisms; and by altering the effects of mechanisms on impact.

**Table 2.** Actions interpreted as ways of determining or changing attributes of treatments to reduce the adverse effects on biodiversity of mechanisms, as influenced by moderators (see [26] for more detail).

type of variable in causal path (figure 4)	related actions
*Treatments* are aspects of the type and management of protected areas, defined by attributes. Examples of attributes are extent, legal status, allowed and regulated uses, community participation, type of enforcement, and arrangements for sharing of revenues.	allocate or change attributes of the treatment to reduce the adverse effects of mechanisms on biodiversity, e.g. define legal status as strict or multiple-use, improve boundary demarcation, or increase funding for surveillance to reduce poaching.
*Mechanisms* respond to treatments. Their values under protection, when compared with counterfactual values, determine impact. Examples are densities of invasive plants, access to exploitable forest, and lack of compliance due to conflict with protected-area managers.	allocate or change attributes of treatments to reduce values of mechanisms that are threats to biodiversity.
*Moderators* are variables that modify the effects of the treatment, but are not affected themselves by the treatment.	allocate or change attributes of the treatments to mitigate or enhance the effects of moderators.
Moderators can precede the establishment of protected areas, e.g. extinction debt owing to historical fragmentation of native vegetation in a region of conservation interest.	choose attributes to mitigate adverse effects of pre-existing conditions on the treatment's impact, e.g. reduce hunting of extinction-prone species, translocate individuals to maintain abundance.
Moderators can determine what mechanisms and what levels of those mechanisms arise from a treatment, e.g. the presence of endangered aquatic species could constrain the influence of a treatment on the mechanism ‘terrestrial weeds'.	choose attributes to enhance the positive effects of the treatment on the mechanism, e.g. invest in physical weed removal or alternative herbicides with less impact on aquatic organisms, or schedule weed control to coincide with time of least sensitivity of aquatic species.
Moderators can constrain the influence of a mechanism on impact, e.g. extensive clearing around a protected area after its establishment leading to isolation (extinction debt) and cross-boundary threats, reducing the impact derived from reducing the value of the mechanism ‘hunting’.	choose attributes to mitigate the adverse effects of the moderator on the mechanism's impact, e.g. invest further in prevention of hunting, maximize internal suitability for species of concern.

The upshot of the framework in figure 4 is that practitioners’ levers are choices about attributes that determine treatments with the aim of reducing values of mechanisms (threats) and enhancing or mitigating the (positive or negative, respectively) effects of moderators on impact (table 2).

We focus on six variables that are widely discussed [and see 67] as influencing the effectiveness of protected areas (figure 4). Of the six, two are aspects of treatment (management intent and management effectiveness), the extent of protected areas is an attribute, and three are moderators (extinction debt, permeability of boundaries, and location). For simplicity, figure 4 shows the two treatments as strict protected areas and highly effective management (with a generic set of attributes in addition to extent) in contrast to no protection, with impact measured as differences in estimates of forest cover and persistence of species with and without protection. Also for simplicity, we show mechanisms as generic 'threats within protected areas' or 'threats without protection'.

### (a) Treatments, attribute, moderators

#### (i) Management intent

Our first treatment is the management intent of protected areas (figure 4). A key consideration here is type of protection, most often interpreted in relation to IUCN (the World Conservation Union) categories that range from strict preservation to multiple-use [1]. Informal protected areas, which might or might not align with IUCN categories, are also extensive and important [68,69]. Management intent influences impact via restrictions on extractive uses. In areas under threat, all other things equal, stricter protection would have more impact, so practitioners could increase impact by altering both the IUCN categories and within-category details of regulated and allowed extractive uses. In practice, other things are not equal: management intent affects where protected areas are placed, what attributes of the treatment are implemented and the values of mechanisms in the face of, for example, levels of conflict with current or aspiring users of natural resources [26]. Management intent in relation to IUCN categories can also be obscured, even in countries with generally strong governance, by tacit or explicit weakening of protected areas that opens them to previously unintended extractive uses [70] or reduces their alignment with IUCN definitions [71].

#### (ii) Management effectiveness

Our second treatment—management effectiveness (figure 4)—refers to the support available for management intent. Management of protected areas is pervasively under-funded [19,72], but management effectiveness is a composite index, not only of inputs for staffing, infrastructure and equipment, but also of aspects of governance, managerial systems, training, capacity for enforcement, liaison, communication and other variables [19]. Constraints on management inputs limit monitoring of protected areas [19], the basis for adaptive management [73]. Compliance with regulated uses by people seeking to extract resources from protected areas can depend strongly on management effectiveness (although also on other variables under the control of managers [74]). With strong governance, the legal existence of protected areas is often sufficient to prevent incursions for extractive uses, at least where detection is likely and the consequences of breaches are serious. However, illegal extraction can occur even in well-enforced protected areas [75]. Where governance is weak, protected areas might be willfully downgraded [76] or suffer frequent and extensive incursions that threaten species and ecosystems [77,78]. Compliance aside, management effectiveness is relevant not only to reducing the effects of threats from outside protected areas, but also to interventions in processes that arise from within protected areas themselves. The persistence of some species under protection will depend, for example, on the regulation of appropriate disturbance regimes [79,80] or of abundances of other native species that are predators or competitors.

#### (iii) Extent of protected areas

We interpret extent as an attribute of our two treatments (figure 4), analogous to the ‘dose’ of protection allocated within a region. The biodiversity impact of a protected-area system depends on the impact of its individual protected areas, which can vary markedly from one to another [16]. In turn, the impact of any single protected area depends on the impact of its smaller parts, which can also be spatially variable [81,82]. The influence of extent on overall impact will depend on the type of impact being considered. For avoided loss of forest cover, the effect of extent might be simply multiplicative—the average per-unit-area impact times the extent of protected areas—unless extent interacts with shape or location [83] to regulate access for illegal extraction. For the persistence of species, the moderating effect of extent will depend also on the species–area curve (the nonlinear accumulation of species as different ecosystems are increasingly represented in protected areas [84,85]) and emergent properties of protected-area systems, such as connectivity,^3^ that will influence extinction debt, below.

#### (iv) Extinction debt

An important effect of extinction debt is to modify the influence of mechanisms on impact measured in terms of the persistence of species. Most terrestrial protected areas owe an (often increasing) extinction debt, defined as ‘time-delayed but deterministic extinction’ or the ‘future ecological cost of current habitat destruction’ [86, p. 65]. Extinction debt has not only been recognized widely in terrestrial environments, but also applies in principle to marine and freshwater ecosystems [87]. On land, the primary reason for extinction debt is extensive and mounting loss or alteration of unprotected native vegetation cover, leading to isolation and insufficient size of protected areas and negative edge effects [35,63,88]. This means that ongoing loss of species from protected areas [89] will to some extent counteract the increasing number of species represented as protected areas expand. Importantly, though, losses of species from protected areas can be mitigated. They might be reduced by choosing attributes related to restoration and management both within protected areas [90] and in their surroundings [91] and by careful design of new protection. Extinction debt is an example of cross-boundary interactions between protected areas and their surrounds, the effect in this case operating from outside to inside. Interactions in the reverse direction are also relevant to impact evaluation.^4^

#### (v) Permeability of boundaries

Another moderator also illustrates cross-boundary interactions: the permeability of protected-area boundaries to some external threats (figure 4) that exacerbate current and future losses of ‘protected’ species [61,97] and can affect vegetation structure and extent. Leaving aside direct incursions by people (affected by the treatments above), examples of threats that cross protected-area boundaries are invasive species [98], domestic animals, polluted run-off [99], altered stream flows [100] and climate change [101]. We interpret this moderator as influencing two parts^5^ of the causal chain between the treatment and resulting impact (figure 4). First, post-protection intrusions of threats will determine the value of mechanisms, such as density of weeds, arising from the treatment. Second, for intruding threats that cannot be eradicated internally or stopped by other means, ongoing effects within protected areas will modify how mechanisms lead to impact. Both influences could be mitigated by choosing attributes that involve, for example, active management interventions inside protected areas, regulation of off-park activities, liaison with owners, managers or users of surrounding areas and strategic modification of boundaries, perhaps along catchment divides [102].

#### (vi) Location

Our third moderator is the location of protected areas. Location is an amalgam of different moderating variables, especially those, such as soil type, slope, rainfall and density of commercial species, that determine the cost, feasibility and political attractiveness of protection. We focus here on location to pre-empt threats^6^—reflecting the primary role of protected areas—either before threats arrive or to mitigate threats where they are already established.

Like other moderators, location can affect the values of mechanisms arising from treatments and the influence of mechanisms on impact (figure 4). Together, these effects could mean, for example, that strict protection on economically valuable land might be only partly successful in reducing incentives for exploitation, perhaps leading to lower impact than would have been achieved with less strict protection or location in less threatened areas [104].

Location also influences the choice of attributes that determine the protected-area treatment (figure 4). Here, it can be useful to distinguish two kinds of threats to biodiversity: those arising directly from extractive uses, such as clearing of native vegetation, logging, grazing, hunting, mining and commercial fishing; and other threats, sometimes related indirectly to the first type, such as invasive species, inappropriate disturbance regimes and polluted run-off.

Protected areas tend to be located in areas least threatened by extractive activities and, arguably, least in need of conservation intervention. This widely recognized, non-random pattern on land [13], now emerging in the sea [12], is a confounder to consider when estimating impact [14,81], as well as the principal reason for low impact of protected areas. The confounding effect will apply also if extractive threats modify the type of protected areas chosen [12] and their management regimes. High extractive threat will probably require large investments in enforcement, for example.

In relation to threats not arising directly from extractive activities, agencies and NGOs can take several possible approaches to establishing protected areas: (i) focus on low-threat areas, regardless of the loss of species and ecosystems elsewhere (the dominant approach in relation to extractive threats); (ii) where there are spatial options for the conservation of particular species and ecosystems, protect the lower-threat examples; and (iii) where there are no options—the only places for protection of certain species and ecosystems are already threatened—applying protection to mitigate established threats. There is little information on which approaches are taken but, if organizations seek primarily to minimize management liabilities, they will favour the first two, causing location relative to threats to confound estimates of impact (figure 4).

### (b) Three implications of this analysis of treatments, attribute and moderators

At the beginning of §3, we indicated that our analysis of treatments, attribute, and moderators had three implications for policy and practice. We now explore those implications.

#### (i) Implication 1: poor evidence base for ways of increasing impact

Because our treatments, attribute and moderators are widely acknowledged as influencing the effectiveness of protected areas, a quick review of the evidence for their relationships with impact is warranted. The evidence is weak for the links between impact and our two treatments. Any relationship between impact and management intent is clouded by the complex ways in which the effects of intent are mediated (in relation to management effectiveness, via mechanisms and moderators, subject to confounding variables). One result of this complexity is that stricter protection does not necessarily lead to more impact [15,16,105,106]. Further complicating the picture are adjustments by governments of regulated and allowed uses that do not align with definitions of IUCN categories, increasing the number of treatments to be evaluated. Overall, there is a poor understanding of the situations in which different management intents, including treatments outside IUCN's protected-area categories, produce most impact in relation to the costs of establishing and managing protection.

Management effectiveness presents a problem for practitioners. On the one hand, the assessment of management effectiveness has enormous momentum globally. It is a major activity of IUCN's World Commission on Protected Areas, with thousands of assessments completed [19,65]. On the other hand, there is no evidence that management effectiveness is related to impact [65]. Before this treatment can be employed to improve protected-area impact, considerable work is needed to establish causal links and understand the mechanisms and moderators that shape these links [26]. For example, management effectiveness in reducing illegal extraction from protected areas might make little difference in strongly residual places, but could have a large impact where protected areas are suitable for agriculture, logging and hunting.

The effects on impact of our attribute and three moderators, taken individually, indicate ways forward for practitioners. We can summarize their individual effects as follows, using the examples of forest cover and persistence of species from figure 4.

— The links between extent and impact are tenuous (figure 4, and see §3b(iii)). Increasing the total extent of protected areas will tend to increase their overall impact, although the effect, linear or otherwise, might be slight if per-unit-area impact is very small, as observed for forest cover [14,15]. The key, then, is to increase extent in parallel with per-unit-area impact, not alone.
— The undesirable moderating effects of extinction debt can be mitigated by management within protected areas (e.g. reintroduction of lost species; maintenance of conditions conducive to species likely to be lost), off-park protection and restoration of conditions outside protected areas to reduce risk of loss, and location and configuration of new protected areas to minimize future losses.
— The undesirable moderating effects of permeability of protected-area boundaries can be mitigated with the same approaches as above.
— Location is potentially a very influential moderator (and see §3b(ii)), but harnessing its capacity to enhance impact would require a reversal of the pervasive residual approach [9–11] to locating protected areas.

How these variables operate in combination is much less clear. Their relative importance in determining the impact of protected areas, and the relative benefits of addressing them to increase impact, are likely to vary strongly across protected areas and, in some places, over time.

Planners and managers will be better at maximizing the impact of already-established and new protected areas when they better understand how to combine actions under their control (table 2). There are several ways forward (adapted from [26]):

(1) Develop theories of change for different kinds of protected areas and management regimes (treatments, defined by attributes) in different ecological, physical and socioeconomic contexts (moderators). The theories of change, beginning with basic frameworks such as figure 4 here and figure 3 in [26], would summarize knowledge and assumptions about the ways in which actual or potential actions (modifications of treatments, targeting of moderators) affect ecological and social mechanisms to achieve impact.
(2) Elaborate on theories of change by estimating the relative importance of different actions (table 2) in increasing the impact of protected areas in different contexts. Insights into investment strategies for combinations of actions could be gained from qualitative models, statistical models and process-based models of protected areas interacting with their social and ecological contexts [107].
(3) Adapt the quasi-experimental designs applied to estimating protected-area impact from observational data to draw inferences about the influence of actions on impact. Three approaches, varying in rigour, have been proposed [26].
(4) Manipulate actions in established protected areas experimentally, with designs intended to isolate the influence on impact of individual actions [108].

The medium- to long-term goal of these approaches is to develop a body of evidence and expertise that will progressively improve protected-area planning and management [109]. Decisions will need to be made, of course, in the meantime, but investments in protected areas will be more effective and accountable as the evidence base moves from step 1 to step 4 in the list above.

#### (ii) Implication 2: location sets the ceiling for impact

**Figure 5.**
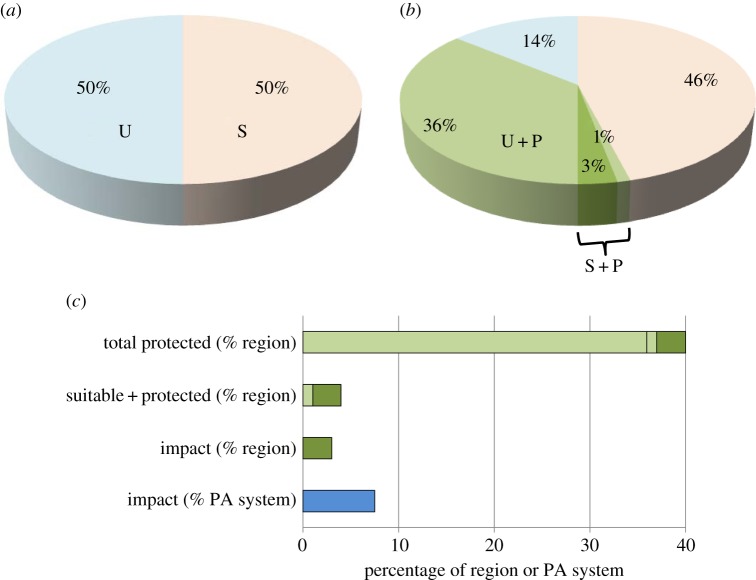
Contribution of protected areas to impact in a hypothetical forested region. In (*a*), U and S indicate land unsuitable and suitable, respectively, for agriculture that involves conversion of forest. In (*b*), U + P and S + P indicate unsuitable and suitable land, respectively, that has been placed in protected areas. Part (*c*) contrasts the overall extent of protected areas with impact expressed as percentages of the region and the protected-area system.

One of the moderators discussed above—location of protection—can determine the upper limit of protected-area impact in a region. To illustrate, in a hypothetical forested region, half the land is suitable for agriculture and half is unsuitable (figure 5*a*). Protected areas are strict (no extraction) but largely residual (figure 5*b*), concentrated on unsuitable land. Protection of unsuitable land amounts to 36% of the region. Protection of suitable land is much smaller, amounting to 4% of the region. The remainder of the suitable land has either been cleared for agriculture or will be in the absence of protection. Of the suitable land protected, a quarter of the forest cover has been lost post-protection to illegal incursions and transformed into agriculture, but attributes related to compliance are sufficient to secure the remaining area as protected (3% of the region). The impact of the protected areas, measured as avoided deforestation, is 3% of the region: the loss to agriculture that has been avoided. This impact amounts to 7.5% of the protected-area system (figure 5*c*), similar to the amount estimated for Costa Rica [14], and larger than estimated in other rigorous studies based on loss of native vegetation [13,15,16].

In this example, per-unit-area impact is heterogeneous within the protected-area system, as observed empirically [16,81,82]. In one small portion of the system, location determines the maximum upper bound of impact (4% of the region): the amount that would have been achieved with full compliance. Investments in higher enforcement and other determinants of compliance [74] could only have reached, not exceeded, this ceiling. The ceiling would be higher only if protected areas covered more of the land suitable for agriculture.

**Figure 6.**
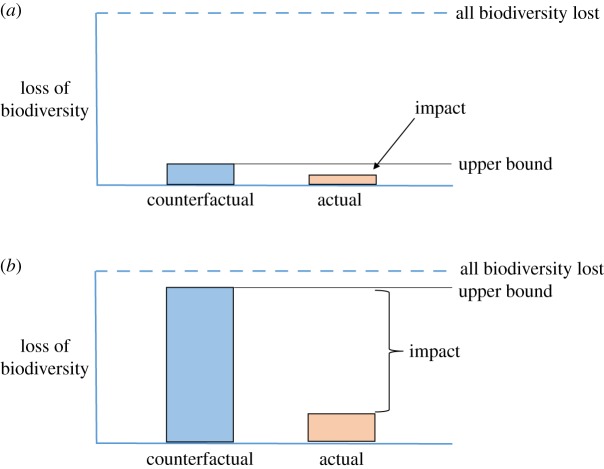
Influence of location of protected areas on impact. Blue bars indicate counterfactuals for loss of biodiversity, setting the upper bounds of impact. Actual impact is the difference between actual loss of biodiversity (orange bars) and the upper bounds. (*a*) Location of protection in area with little threat to biodiversity, counterfactual loss low, impact small. (*b*) Location of protection in area with high threat, counterfactual loss high, impact large. In these examples, actual losses (orange) can be seen as the losses that protection failed to avoid, e.g. owing to insufficient management resources to eliminate threats from outside.

Figure 6 provides another perspective on location. The counterfactual result sets the upper bound on the impact that fully effective protection can achieve in any area, and the counterfactual result is determined by threats. The upper bound on impact is small where threats are low (figure 6*a*) and large when threats are high (figure 6*b*). Locating protected areas relative to threats therefore determines the maximum impact that can be achieved (and see [15,16] for empirical examples). Once a decision is made on location, how much of the upper bound on impact is realized will depend on how close to ‘fully effective’ (full compliance, no loss of biodiversity) the protected area becomes, as determined by attributes, treatments, mechanisms and changes to the effects of moderators (figure 4). Investments and decisions after location have been fixed cannot extend impact beyond the upper bound. Another consideration here is that location, once fixed, is much more difficult to change than actions within protected-area boundaries. The pervasive tendency to locate protected areas where threats are low [9–11] has been a major constraint on impact. Deliberately enhancing the impact of protected-areas will rely partly on enhancing the impact of established ones but largely on locating new ones to pre-empt or mitigate threats, not to sidestep them.^6^

#### (iii) Implication 3: extent of protected areas is a poor guide to impact

The extent of protected areas is an attribute of protected areas that converts per-unit-area impact into the overall impact of a protected-area system. The relationship between extent and impact is, however, very indirect, depending on other attributes and many intermediate variables (figure 4). If average per-unit-area impact is small, then extent is a poor guide to performance. Extent can be confused with impact by assuming that, in the absence of protection, all biodiversity would be lost. Applied to figure 5, the corresponding assumption would be that biodiversity in all 40% of the region protected would be lost without protection. This impressive extent of the protected areas, however, greatly over-estimates their impact (figure 5*c*) because it fails to consider what would actually happen in the absence of protection. The actual impact in figure 5-much smaller than the extent of protection–is realistic, even generous, compared with empirical estimates [13–16].

The examples in figures 5 and 6 add to our discussion of outputs in §2. Extent of protected areas is simply not a reliable guide to how much loss of biodiversity has been avoided. Commonly used targets and measures of progress that depend on the number, total extent or percentage coverage of protected areas [50,110]—outputs in figure 1*a*—are therefore misleading in terms of the fundamental purpose of protection.

## 4. Shifting policy, planning and management towards protected-area impact

Here, we outline feasible steps towards policy targets that define a vision for protected-area impact. We also outline feasible approaches to planning and management of protected areas directed towards impact. Our outlined approaches are far from comprehensive, but are intended to contribute to, and hopefully stimulate, much-needed discussion about these topics.

We shift here to a model (figure 1*b*) in which policy targets and operational objectives for planning and management are directed explicitly to impact. In this model, outputs and outcomes for sampling are reported, but are not ends in themselves, and are not necessarily maximized. Estimates of impact are based on explicit counterfactual thinking and quantitative methods [2], noting that the reliability of impact estimates varies with study design and quantitative rigour [14].

We make five general points here to establish the context for moving towards improved targets and objectives. First, policy-makers and protected-area practitioners must decide exactly what they intend to achieve. Although measures of impact related to threats might have some advantages over those based directly on biodiversity [26,28,58], they come with risks. Maximizing impact in relation to threat, for example, will not necessarily maximize impact in relation to biodiversity, depending on the threats, species or ecosystems in question (see §2b(iv)). Also, a focus on threats could direct protection to places where threats are most easily avoided, not where benefits to biodiversity are greatest. Where possible, therefore, policy targets and planning objectives are best related to biodiversity, and we take this approach below (so ‘avoided threats’ in figure 1*b* is a means to the end of avoided loss of biodiversity, not an end in itself).

Second, all measures of impact are to some extent proxies. A positive impact in terms of avoided deforestation, for example, does not necessarily mean a positive impact for all ecosystems and species. In any case, ecosystems are inevitably proxies for species or other features of primary interest, and measurement of impact is only possible for a few, better-known species. Similarly, avoided deforestation is not necessarily a good proxy for avoided release of carbon [111]. The limitations of proxies should therefore be remembered in evaluating impact, but are no greater than in other aspects of policy, planning or management [112].

Third, more effective policy, planning and management will rest on prediction of protected-area impact. Almost all impact evaluations have been retrospective, helping to guide improvements for protected areas already in place and providing lessons for future decisions, but future decisions must be informed by spatially explicit data on where most impact can be achieved. Predictive studies [43,104,107,113,114] provide the foundation for refined approaches that will need to integrate forward-looking models with lessons from established protected areas.

The fourth point concerns the difficulty of reversing decisions about the locations of protected areas, and therefore, the importance of getting location right to begin with. Although some protected areas are degazetted, reduced or downgraded for purposes other than enhancing nature conservation [115], strategically removing and replacing them to improve impacts for biodiversity [116] come with severe practical constraints.

Our final point concerns the short-term ability to refocus policy, planning and management towards impact. An immediate constraint on moving forward is the weak evidence base relating impact to the actions (table 2 and figure 4) that planners and managers can take. In §3b(i), we laid out steps needed to improve this evidence, beginning with theories of change, then extending to models of the relative importance of actions and finally to quasi-experimental and experimental designs. In the sections that follow, we refer to these steps as ‘models for taking action’. Our generic term ‘actions' refers again to the attributes that define treatments and alter the effects of moderators (table 2).

### (a) Towards policy targets to better achieve impact

Maximizing protected-area impact will rely to a large extent on the global direction and motivation provided by aspirational targets from international organizations (especially IUCN's World Commission on Protected Areas) and multi-lateral environmental agreements such as the Ramsar Convention (http://www.ramsar.org/) and the Convention on Biological Diversity (https://www.cbd.int/). At present, these aspirational targets are focused almost solely on parts of the results chain in figure 1*a*, with the serious risk of misdirecting conservation efforts.

Among the Convention on Biological Diversity's Aichi targets [17], very few are unambiguously quantitative. One of them—Target 11—requires 17% of land and 10% of the sea to be in protected areas by 2020, but is solely concerned with outputs, so does not touch on impact. Another one—Target 5—potentially concerns impact, but in only vague, qualitative terms: ‘By 2020, the rate of loss of all natural habitats, is at least halved and where feasible brought close to zero, and degradation and fragmentation [are] significantly reduced’. The present utility and influence of Target 5 are constrained by lack of information on the identity of ‘habitats', baseline rates of loss, the starting date from which the halving should be accomplished, and which habitats should have rates of loss closer to zero. Another constraint is the lack of guidance on how to interpret ‘significantly reduced’ degradation and fragmentation. More fundamentally, though, Target 5 fails to define the counterfactual against which protected areas are meant to make a difference. One interpretation of Target 5 is that halving rates of loss is a performance measure—an outcome (figure 2) that might, or might not, be attributable to protected areas themselves. Reductions in loss rates might arise also from conservation outside protected areas, changes in market forces, or some habitats having been almost completely removed [117]. A second interpretation of Target 5 is that the baseline rate of loss is the counterfactual: the baseline rate of loss is the expected future rate of loss to 2020 in the absence of additional or strengthened protected areas. This counterfactual would be unreliable, given predicted between- and within-country changes in rates of loss between 2015 and 2020 compared with previously [43]. Neither of these interpretations is satisfactory, highlighting the need for formulation of a new protected-area target to address impact explicitly.

A protected-area target under the Convention on Biological Diversity that explicitly addresses impact will need to be supported by at least the decisions and data listed in table 3. In outlining this approach, we acknowledge that many decisions about protected areas will not take a global perspective. The steps in table 3 could be applied within national, regional, or local contexts. Much of the value of the required global leadership will be its motivation for decision-makers to achieve impact in any spatial context, and to avoid the distraction of targets in the results chain of figure 1*a*.

**Table 3.** Steps towards a protected-area policy target to better achieve impact.

goal	strengthen and expand protected areas to achieve a specified amount of impact (we use this goal as an example here, although the steps below could be adapted to an alternative goal of maximizing the impact of a specified extent of strengthened and new protected areas)
data	decide how impact will be targeted and measured. The most meaningful options will be avoided loss of ecosystems and/or species, in terms of, for example, extent, abundance, or likelihood of extinction. If species distributions are to be used, decide on acceptable types and quality of data. If ecosystems are to be used, decide on an approach to define them spatially
	agree on definitions of ‘loss’, ‘degradation’ and ‘fragmentation’, in terms of change detectable with spatial and temporal consistency from remote sensing or other data sources
counterfactual(s)	use spatially explicit modelling to project future loss of species and/or ecosystems in the absence of strengthened and additional protection [43]. Explore uncertainty around the patterns and trajectories of loss with multiple scenarios
upper bounds	for each species and/or ecosystem, estimate the upper bounds of impact (figure 6) that could be achieved. Within established protected areas, the upper bounds (likely to vary between species and ecosystems) will be the differences between estimated values without protection and estimated best-possible values with fully effective protection (table 4). For areas outside protection, the upper bounds will be the differences in distribution or abundance between the counterfactual and present values
targets	decide on the time interval over which targets should be achieved
	considering the upper bounds on impact, specify the amount of impact that should be achieved and how it should be distributed across each species and/or ecosystem
actions and costs	there are two alternatives here. One is to decide how much of the specified amount of impact will be achieved by strengthening existing protected areas versus locating, configuring, and managing additional protected areas. The second is to let this balance emerge from a prioritization process that allocates actions to achieve the specified amount of impact at minimum cost
measures	decide on methods for tracking progress for individual species and/or ecosystems
	decide on aggregate measures of progress across species and/or ecosystems, considering factors such as cost-effectiveness, reliability in reflecting status and trends, responsiveness to policy changes, and mathematical properties [118–120]

### (b) Towards planning and managing protected areas to better achieve impact

The impact of a protected-area system results from hundreds or thousands of accumulated choices by planners, managers, organizations and governments. The small impact of protected-area systems could therefore be seen as an expression of ‘the tyranny of small decisions' [121]: a big decision occurs (post hoc) as an accretion of many small decisions, without the central question being addressed directly. As far as we know, no one purposefully set out to produce a global protected-area system with little impact, but that is what we have.

Ways of making individual decisions that accumulate to increase overall impact are outlined here. The strategy will combine improvements in the impact of existing protected areas with establishment of new protected areas directed at achieving impact.

For improving the impact of established protected areas (table 4), the spatial relationship between protected areas and existing threats or emerging and intensifying threats has already been determined. For new protected areas, location is a potentially important moderator that planners and managers can influence (and see §3b(ii)). For new protected areas, goals can also be stated in different ways: protected areas can be expanded to achieve a specified amount of impact (the example here), or the impact of a specified extent of new protected areas can be maximized. For our example goal, the outlined steps are similar to those for a global policy target (table 3) but with this large picture scaled down within regions or jurisdictions to a spatial resolution as close as possible to that of decisions on the ground or in the water. Decisions about where and how to protect areas to achieve impact would also need a more nuanced assessment of the costs and returns of specific actions in combination.

**Table 4.** Steps towards managing established protected areas to better achieve impact.

goal	maximize the impact of established protected areas subject to a total management budget (the steps below could be adapted to an alternative goal of strengthening established protected areas to achieve a specified amount of impact)
data	decide how impact will be targeted and measured (refer to table 3)
	for logistical reasons, restrict the scope of the problem to ‘high-priority’ protected areas. Identify these protected areas by estimating the contribution they make to conservation objectives ranging, in order of importance, from global to subnational and local
counterfactuals	for each protected area, use models for taking action to estimate the counterfactuals, that is, the distribution or abundance of selected species and/or ecosystems in the absence of protection
upper bounds	for each protected area, use models for taking action to estimate the distribution or abundance of selected species and/or ecosystems with fully effective protection. Then subtract the counterfactual estimates to get the upper bound of impact that could be achieved (figure 6)
objectives	decide on the time interval over which objectives should be achieved
	for each protected area and each selected species and/or ecosystem, considering the upper bounds on impact, specify the maximum amount of impact that should be achieved (revise as necessary, subject to below). We assume that the selected species and/or ecosystems will tend to differ between high-priority protected areas
actions and costs	for each protected area, use models for taking action to identify the actions needed to achieve the specified maximum amount of impact. If actions will not achieve the specified amount, revise that amount
	estimate the costs of taking the required actions, and whether the cost-effectiveness of actions is likely to diminish with higher amounts of impact achieved. If costs of actions produce diminishing returns with higher amounts of impact, consider whether the specified amount of impact should be reduced and actions partly reallocated to other protected areas. If so, revise the amount of impact to be achieved
	allocate actions to protected areas to maximize the achievement of objectives, and implement those actions
measures	decide on methods for tracking progress for individual species and/or ecosystems
	decide on aggregate measures of progress across protected areas and across species and/or ecosystems (refer to table 3)

## 5. Challenges and opportunities

Policy-makers, planners and managers are focused largely on performance measures that do not reflect the basic purpose of protected areas: to make a difference. If protection is to be more effective in reducing the loss of biodiversity, impact must be central to policy and operational planning and management.

Decision-making processes must be recast around policy targets and operational objectives for impact. The key requirements for a revised policy target, building on the example in table 3, are already in place: available data, modelling capabilities and the capacity to coordinate global activities. At the operational level, it is also possible to design decision-making processes to increase impact (table 4). However, progress in planning and management for impact, although urgent, is limited by poor understanding of what combinations of actions will best lead to impact in particular situations.

A commitment to increasing protected-area impact will require decisions and investments while the evidence base for the required actions is being assembled. We have outlined four ways in which data and experience can be organized systematically to guide actions that support impact, increasing in rigour from theories of change to experimental designs. Policy-makers and practitioners should expect that these methods, like impact evaluation more broadly, must become standard practice for decisions about protected areas [4], just as they are in other fields, such as medicine and development aid, in which the consequences of wrong decisions are serious and irreversible.

The decision-support tools needed to guide policy and operational decisions to increase impact are either available or within reach. Tools for spatially and temporally explicit modelling of land use change [43] and process-based models of ecosystem responses to management [107], for example, can be applied and refined to estimate the counterfactuals needed for predicting impact. The process models can also be used to help understand how actions can be combined to increase impact [114]. Conservation planning software is not yet adequate for achieving impact (table 4), but developments are underway to handle, for example, multiple actions in any area, different levels of specified actions (recognizing nonlinear relationships between costs and effects), and graphical user interfaces to facilitate interactive use.

The scene is set for a shift in policy and practice to realize the full potential of protected areas. How soon and how effectively this shift is made will depend on leadership from global organizations and conventions and on proof-of-concept projects by agencies and NGOs. Every year of delay means irreversible, avoidable loss of biodiversity.

## Endnotes

1 We use the term here as shorthand for any spatial depictions of physical and ecological composition, including habitats, vegetation types, environmental domains and ecoregions.

2 We use the term generically to refer to any theoretical analysis of the causal chain linking interventions and the results that they produce. So the term applies to the results chain in figure 1*a* (which does not include impact) and the causal chains leading to impact in figures 1*b*, 3 and 4.

3 In the kind of causal analysis illustrated in figure 4, connectivity could be an attribute, a moderator, or both. It would be an attribute if the intention of protected-area design were to increase compactness or strengthen links to nearby intact forest. Connectivity would be a moderator if it were determined by events outside protected areas, such as clearing or road construction on nearby private lands, beyond the influence of management.

4 More typically, ‘spillover’ refers to the positive or negative effects of protected areas on areas outside their boundaries. These effects can relate to human responses [92,93]. Negative human effects on biodiversity outside protected areas could arise from attraction of development or settlement for lifestyle or livelihoods, or displacement of pressure for extractive uses. Positive effects could arise from decisions to buffer protected areas with constraints on extractive uses. Other external spillovers are physical or biological, as when water quality is maintained or enhanced in streams originating in protected areas [94] or when marine protected areas subsidise fish biomass and recruitment in unprotected sites [95,96]. These spillovers from within protected areas can be accounted for in estimates of impact [14].

5 There are no boundaries pre-treatment, although choice of boundaries (attribute of the treatment), moderated by location and surrounding human activities (both pre- and post-treatment), will determine the later influence of this moderator (table 2).

6 We acknowledge that an effective strategy to pre-empt threats will deal with time-lags and displacement, which we do not cover here. By time-lags we mean that locating protection needs some balance between imminently threatened areas and those that are presently secure but might become threatened later [103]. By displacement, we mean the potential for protected areas to shift extractive pressures elsewhere, increasing the threats faced by some unprotected areas [60]. Neither time-lags nor displacement effects are well understood, and improved methods for modelling both are urgently needed if the impact of protected areas is to be maximized.
